# Supplementary material for: The Gut Bacteria Dysbiosis Contributes to Chronic Graft-Versus-Host Disease Associated With a Treg/Th1 Ratio Imbalance
Source: Front Microbiol. 2022 Sep 8;13:813576. doi: 10.3389/fmicb.2022.813576 (PMC9493085; doi:10.3389/fmicb.2022.813576)
Supplement: Supplementary file 1 [file Data_Sheet_1.zip › P101SC18090073-01-B1-3-4_result/05.WebShow/taxa_charts_html/charts/uZRZNjElWtANQARbDfmhqGO69BQbMz_legend.pdf]

k__Bacteria;p__Actinobacteria;c__unidentified_Actinobacteria
k__Bacteria;p__Bacteroidetes;c__Bacteroidia
k__Bacteria;p__Firmicutes;Other
k__Bacteria;p__Firmicutes;c__Bacilli
k__Bacteria;p__Firmicutes;c__Clostridia
k__Bacteria;p__Firmicutes;c__Erysipelotrichia
k__Bacteria;p__Firmicutes;c__Negativicutes
k__Bacteria;p__Fusobacteria;c__Fusobacteriia
k__Bacteria;p__Melainabacteria;Other
k__Bacteria;p__Oxyphotobacteria;c__unidentified_Oxyphotobacteria
k__Bacteria;p__Proteobacteria;c__Alphaproteobacteria
k__Bacteria;p__Proteobacteria;c__Deltaproteobacteria
k__Bacteria;p__Proteobacteria;c__Gammaproteobacteria
k__Bacteria;p__Tenericutes;c__Mollicutes
k__Bacteria;p__Verrucomicrobia;c__Verrucomicrobiae
k__Bacteria;p__unidentified_Bacteria;c__unidentified_Bacteria
